# The choroid plexuses and their impact on developmental neurogenesis

**DOI:** 10.3389/fnins.2014.00340

**Published:** 2014-10-24

**Authors:** Pia A. Johansson

**Affiliations:** Institute for Stem Cell Research, Helmholtz Center Munich, German Research Center for Environmental HealthMunich, Germany

**Keywords:** choroid plexus, cerebrospinal fluid, neurogenesis, neural stem cells, cerebral cortex, development

## Abstract

During brain development the neural stem cells are regulated by both intrinsic and extrinsic sources. One site of origin of extrinsic regulation is the developing choroid plexuses, primely situated inside the cerebral ventricles. The choroid plexuses are very active in terms of both secretion and barrier function as soon as they appear during development and control the production and contents of cerebrospinal fluid (CSF). This suggests that regulated secretion of signaling molecules from the choroid plexuses into CSF can regulate neural stem cell behavior (as they are in direct contact with CSF) and thereby neurogenesis and brain development. Here, choroid plexus development, particularly with regards to molecular regulation and specification, is reviewed. This is followed by a review and discussion of the role of the developing choroid plexuses in brain development. In particular, recent evidence suggests a region-specific reciprocal regulation between choroid plexuses and the neural stem cells. This is accomplished by site-specific secretion of signaling molecules from the different choroid plexuses into CSF, as well as brain region specific competence of the neural stem cells to respond to the signaling molecules present in CSF. In conclusion, although in its infancy, the field of choroid plexus regulation of neurogenesis has already and will likely continue to shed new light on our understanding of the control and fine-tuning of overall brain development.

## Introduction

**Figure 1:**
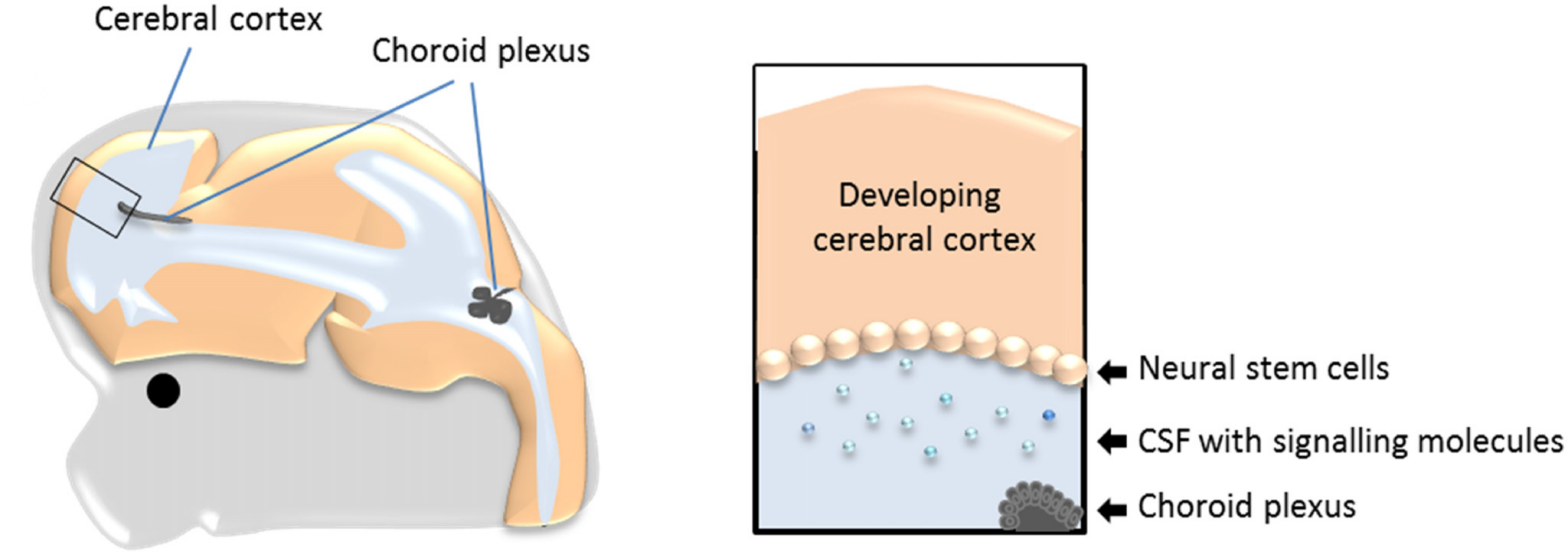
**The choroid plexuses, CSF and the neural stem cells during development**. Schematic drawing showing the prime location of the choroid plexuses inside the cerebral ventricles and the bathing of the neural stem cells with CSF and molecules within it. CSF, cerebrospinal fluid.

Neurogenesis, both in the adult and during development, occurs in a specialized environment established by both the neurogenic (the neural stem cells) and non-neurogenic cells within the neurogenic area, and from cells and compartments in direct contact with the niche (Johansson et al., 2010; Braun and Jessberger, 2014, Figure 1). One of these compartments is cerebrospinal fluid (CSF). During development the neural stem cells are located at the apical (ventricle facing) surface of the developing brain tissue (as exemplified in Figure 1 and Box 1). Thus, any molecule present in CSF has the potential to influence the behavior of the cells located at the ventricular surface (i.e., the neural stem cells) and thereby neurogenesis. The possible role of CSF itself in regulating neurogenesis has recently received more attention, however, there has been less focus on the tissue that produces the CSF; namely the choroid plexuses.

Box 1 **Schematic representation of neurogenesis in the developing cerebral cortex.**

Neurogenesis in the cerebral cortex goes through a differentiation cascade starting with the neural stem cells (NSCs) lining the ventricle (a.k.a radial glial cells, blue cells). The processes of the NSCs span the entire thickness of the cortical wall and their nuclei move up and down in the ventricular zone (VZ) during the cell cycle (mitosis occurs at the ventricle and S-phase at the basal end of the VZ) but they stay in contact with the ventricle via the apical process and the cilium responsive to elements present in CSF through-out the cell cycle. The NSCs divide and give rise to the next cells in the differentiation cascade, the transient amplifying cells, TAPs (yellow cells) or neurons (green cells). The TAPs are a heterogeneous population and vary in their morphology, location and abundance (see Taverna et al., 2014) but in the mouse cerebral cortex the majority of the TAPs are what is known as basal (or intermediate) progenitors which migrated away from the VZ into the subventricular zone (SVZ). The TAPs then divide again and give rise to the neurons which migrate through the intermediate zone (IZ) into the cortical plate (CP) in an inside-out manner (i.e., the newly-born neurons migrate passed the older neurons and settle in a layer above the previous-born neurons, as illustrated by different shades of green). For a recent comprehensive review see Taverna et al., 2014.

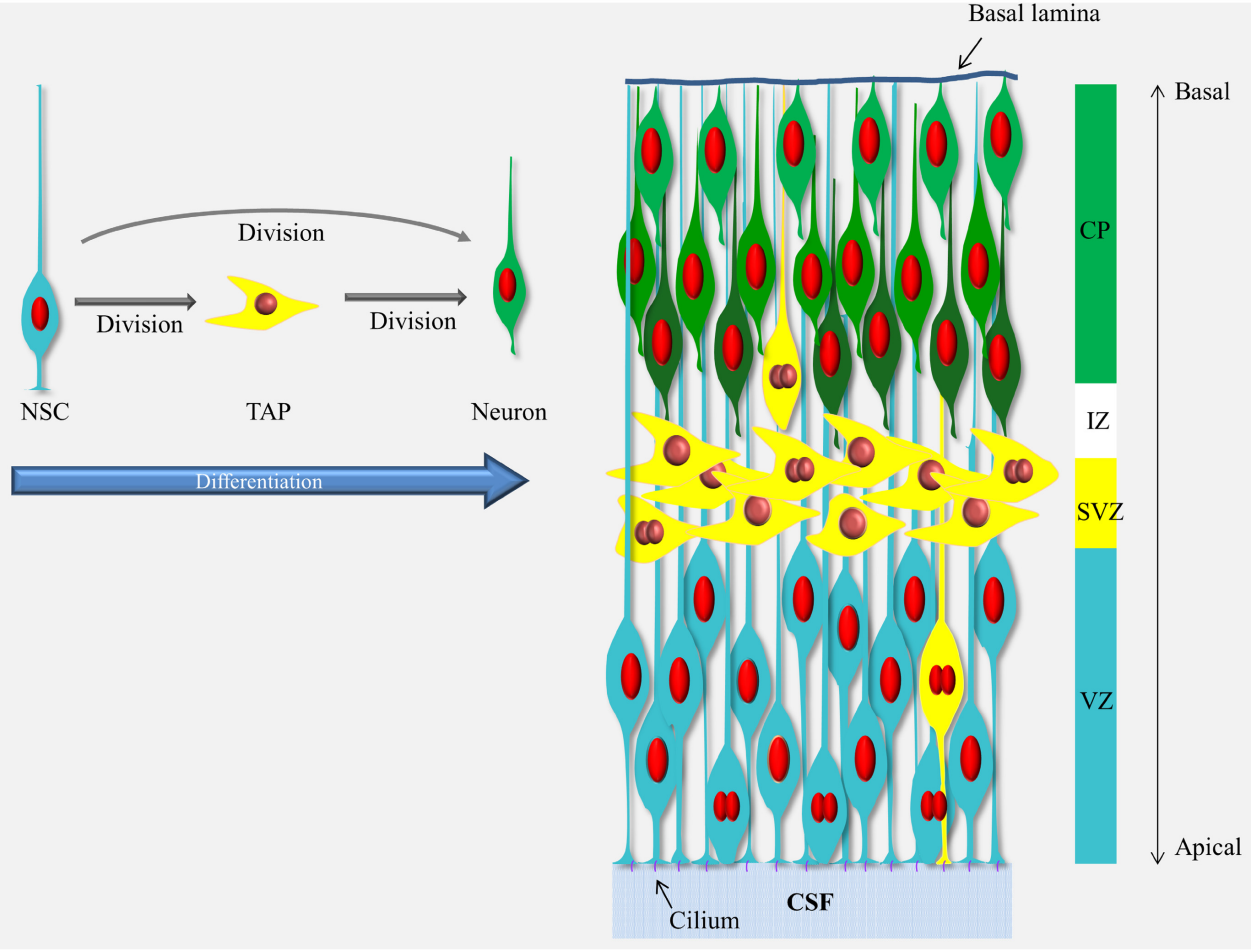

The most abundant proteins in CSF (albumin, transferrin, alpha-phetoprotein, fetuin) are derived from plasma and are transferred across the choroid plexus epithelium by a subset of choroid plexus epithelial cells in a highly specific and regulated manner (Johansson et al., 2006; Liddelow et al., 2009, Liddelow et al., 2011a,b). Over the last decade it has also been shown that in addition to the more classical plasma proteins, CSF contains a variety of comparatively less abundant signaling molecules (e.g., Shh, Igf1, Wnt4, Tgm2, Fgf2, see Table 1). Recent data from transcriptome analysis of the choroid plexus, in both the adult and during development (Marques et al., 2011; Johansson et al., 2013), has revealed the expression of a plethora of molecules previously not associated with the choroid plexus which can either intrinsically affect the function of the choroid plexus or be secreted into CSF. Indeed, a recent comparison of human adult CSF showed that of 1050 identified proteins in plasma 877 of these were also found in CSF whereas an astonishing 2204 proteins were found only in CSF (Guldbrandsen et al., 2014). As CSF is in direct contact with the neural stem cells in both the developing and the adult brain and as CSF is mostly regulated via the choroid plexuses, it is not far-fetched to assume that the choroid plexuses could be significant regulators of neurogenesis.

**Table 1:** **Selected list of secreted signaling molecules expressed in the choroid plexuses**.

**Gene symbol**	**Entrez**	**Gene title**	**Functional annotations/Signaling pathways**
**(A) SECRETED MOLECULES WITH CONFIRMED PRESENCE IN EMBRYONIC CSF AND ABILITY TO INFLUENCE NEUROGENESIS**			
Igf2	16002	Insulin-like growth factor 2	Growth factor activity
Shh	20423	Sonic hedgehog	Shh-signaling
Fgf2	14173	Fibroblast growth factor 2	Growth factor activity
Tgm2	21817	Transglutaminase 2, C polypeptide	Wnt-signaling
Wnt4	22417	Wingless-related MMTV integration site 4	Wnt-signaling
**(B) SECRETED MOLECULES WITH THE POTENTIAL OF INFLUENCING NEUROGENESIS**			
Cthrc1	68588	Collagen triple helix repeat containing 1	Wnt-signaling
Cxcl12	20315	Chemokine (C-X-C motif) ligand 12	Chemokine/growth factor activity
Reln	19699	Reelin	Nervous system development
Dkk2	56811	Dickkopf homolog 2 (Xenopus laevis)	Wnt-signaling
Fgf15	14170	Fibroblast growth factor 15	Growth factor activity
Fgf18	14172	Fibroblast growth factor 18	Growth factor activity
Gdf5	14563	Growth differentiation factor 5	Tgfβ/Hippo-signaling
Pmch	110312	Pro-melanin-concentrating hormone	Neuropeptide signaling
Sema3c	20348	Sema domain, immunoglobulin domain (Ig), short basic domain, secreted, (semaphorin) 3C	Nervous system development
Sfrp2	20319	Secreted frizzled-related protein 2	Wnt/BMP-signaling
Sostdc1	66042	Sclerostin domain containing 1	Wnt/BMP-signaling
Spp1	20750	Secreted phosphoprotein 1	BMP-signaling
Sulf1	240725	Sulfatase 1	Wnt/BMP/FGFreceptor signaling pathway
Tgfb1	21803	Transforming growth factor, beta 1	Tgfβ-signaling
Tgfb2	21808	Transforming growth factor, beta 2	Tgfβ-signaling
Tgfb3	21809	Transforming growth factor, beta 3	Tgfβ-signaling
Wfikkn2	278507	WAP, follistatin/kazal, immunoglobulin, kunitz and netrin domain containing 2	Tgfβ-signaling
Wnt1	22408	Wingless-type MMTV integration site 1	Wnt/BMP-signaling

*Molecules in B from Johansson et al. (2013)*.

So far, only two papers have described a direct link between altered choroid plexus secretion (i.e., via direct choroid plexus manipulation) and alterations in developmental neurogenesis (Huang et al., 2010; Johansson et al., 2013). Hopefully, the recent increased focus on this signaling axis will lead to further contributions to this field over the coming years. Regulation of neurogenesis via molecules secreted (or transferred) from the choroid plexus into CSF could have potentially far-reaching ramifications for the understanding and treatment of both developmental disorders and pathological situations in the adult brain where modulation of neurogenesis might be beneficial. The role of the choroid plexus-CSF signaling axis in adult neurogenesis is beyond the scope of this review but the interested reader is referred to some recent articles and reviews which highlight this interaction (Falcao et al., 2012; Silva-Vargas et al., 2013; Baruch et al., 2014).

## Structure and function of the choroid plexuses

**Figure 2:**
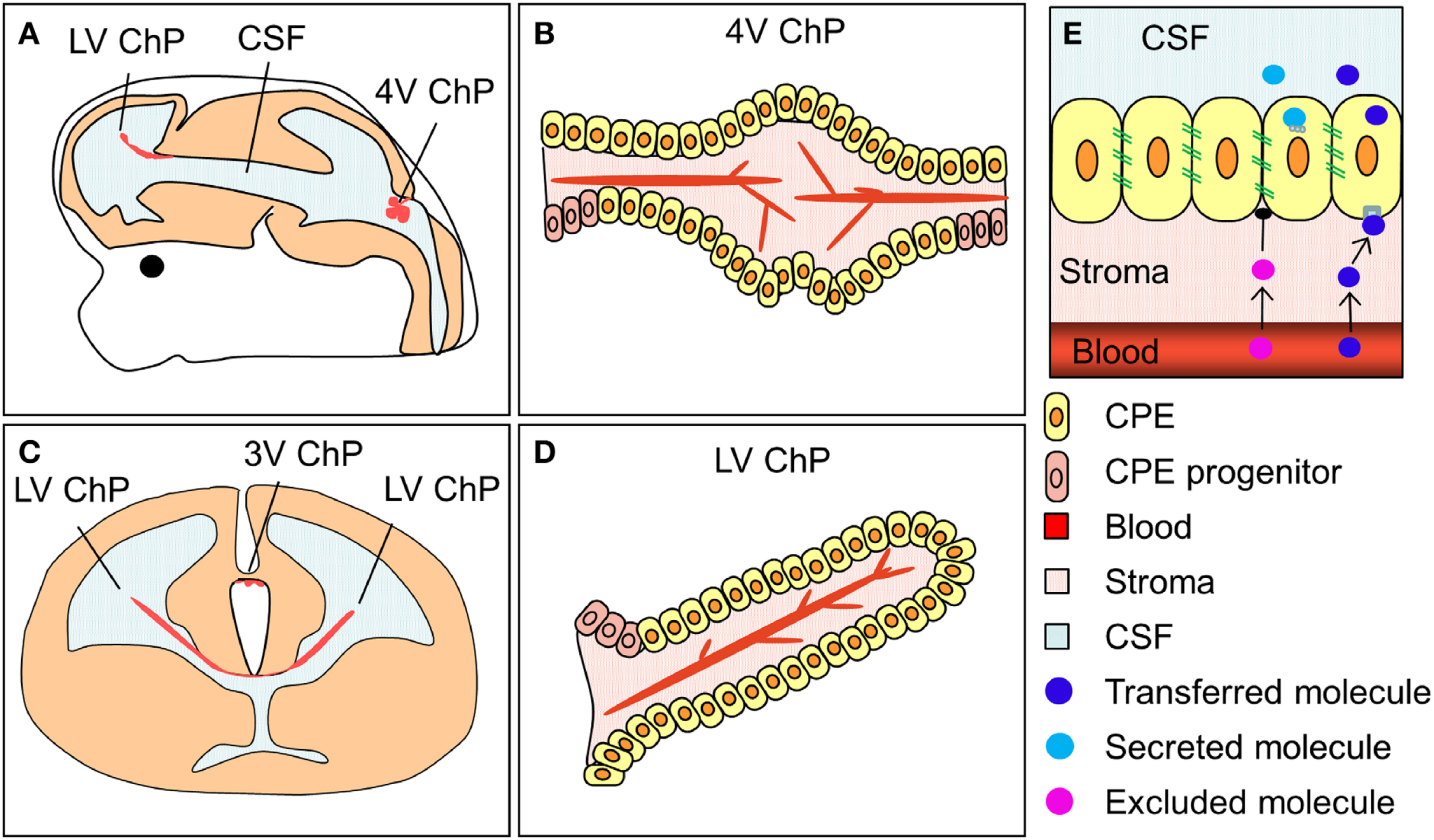
**The location and structure of the choroid plexuses and the blood-CSF barrier. (A,C)** Schematic drawings of an embryonic brain in sagittal **(A)** and coronal **(C)** orientation depicting the location of the lateral, third and fourth ventricular choroid plexuses (LV ChP, 3V ChP and 4V ChP). **(B,D)** schematic drawing of the lateral and fourth ventricular choroid plexuses. **(E)** Simplified schematic depiction of the blood-CSF barrier and the transfer and secretion of molecules into CSF. ChP, choroid plexus.

The choroid plexuses are four modified epithelial structures suspended inside the cerebral ventricles. There are two lateral ventricular choroid plexuses, the third ventricular choroid plexus and the fourth ventricular (hindbrain) choroid plexus (Figure 2). The choroid plexuses consist of a central stroma covered by a single epithelial layer; the polarized choroid plexus epithelium (CPE). The stroma is highly vascularized, consisting of blood vessels, connective tissue and pericytes. The blood vessels are fenestrated and leaky, making them different from blood vessels in the brain parenchyma which are connected by tight junctions and form the blood-brain barrier. Thus, instead of having a barrier at the level of the blood vessels it is the cells of the CPE that are connected by continuous tight-junctional strands, restricting the entry of lipid-insoluble molecules into the CSF (the blood-CSF barrier, Figure 2, Brightman and Reese, 1969; Ek et al., 2003; Johansson et al., 2006).

In the adult, the choroid plexuses and CSF have several know functions such as: (i) protecting and regulating the internal environment of the brain via the blood-CSF barrier; (ii) secretion and modulation of CSF through the activity of the choroid plexus epithelial cells; and (iii) waste and metabolite removal, via the “CSF sink,” through the continuous production and then removal of CSF into peripheral circulation. During development two out of these three functions are already performed by the choroid plexuses; protection of the brain via the blood-CSF barrier and regulation of CSF composition via specific and regulated transfer and secretion (see more below). However, due to the lack of CSF reabsorption during embryogenesis, the removal of waste and metabolites does not occur (Johansson et al., 2008a). There have also long been suggestions for additional roles for the choroid plexuses during development, such as the generation of an expansive pressure through CSF secretion (Desmond and Jacobson, 1977).

## Choroid plexus development

**Figure 3:**
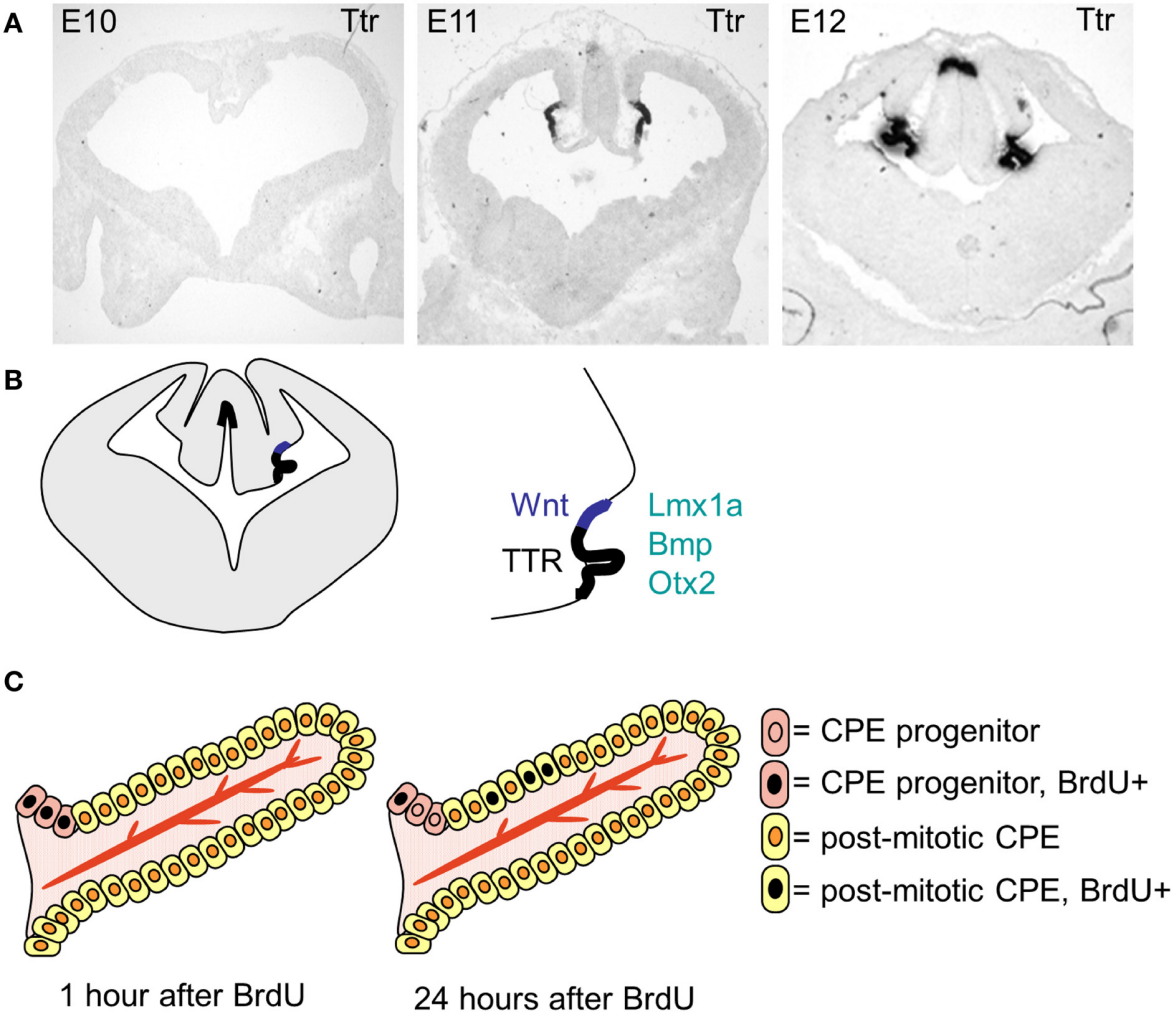
**The specification, differentiation and growth of the lateral ventricular choroid plexuses. (A)** Photographs of *transthyretin (Ttr) in situ* hybridization sections, showing the choroid plexus anlage at E19, E11 and E12. **(B)** Schematic drawing showing the localization of the lateral ventricular choroid plexus and the cortical hem within the invaginated dorsal midline. **(C)** Schematic drawing (modified from Liddelow et al., 2010) showing the localization of the progenitor cells and the mode of growth of the choroid plexus.

The choroid plexuses are of dual embryonic origin, with the choroid plexus epithelium originating from the ectoderm and the central stroma from the mesoderm (Catala, 1998). The fourth ventricular choroid plexus develops first followed by the two lateral and then the third ventricular choroid plexuses (Dziegielewska et al., 2001). In the mouse the dorsal midline starts to invaginate (from dorsal to ventral) around E10, giving rise to the choroid plexus and, in the forebrain, the adjacent cortical hem (Figure 3). The choroid plexus and the hem both express *Lmhx1*, the hem expresses *Wnt* at high levels and the choroid plexus specifically expresses *transthyretin (Ttr)*. *Ttr* is known as *the* choroid plexus marker and is expressed in the mouse lateral ventricular choroid plexus from E11 (Figure 3). However, it should be noted that *Ttr* identifies choroid plexus tissue rather than determining the fate of the cells, as in the Ttr knock-out mice the choroid plexuses form normally.

Other molecules and signaling pathways have been shown to be involved in the specification and formation of the choroid plexus. The inhibition of BMP signaling via Foxg1Cre mediated deletion of Bmp1a receptors led to the absence of the lateral ventricular choroid plexuses (Hebert et al., 2002), however in this study the impact on brain development was not further investigated. A similar result was seen in the developing chick when the transcription factors Emx1 and 2 were mis-expressed in the roof plate region, which lead to the absence of *Bmp7* expression and a failure of the dorsal midline to invaginate (Von Frowein et al., 2006). Interestingly, what was also apparent during the latter experiments was that the transcription factor Otx2 was reciprocally inhibited by Emx1/2, suggesting a role of Otx2 in choroid plexus development. Further investigations into the role of Otx2 in choroid plexus development, this time in mice, revealed that it is indeed vital for choroid plexus specification and development (Johansson et al., 2013). Removal of Otx2 from all the choroid plexus anlage (tamoxifen mediated Cre-induction at E9) resulted in an almost complete absence of choroid plexus tissue. Both the Otx2 and Bmp1a deletion show specific roles for these molecules in choroid plexus development as the nearby cortical hem was unaffected in both mutants.

Another group of molecules involved in choroid plexus differentiation are members of the Notch signaling pathway. When the levels of Ngn2 or Hes5 were altered in the telencephalic midline (over-expressed and down-regulated respectively) the choroid plexus was reduced in size but Cajal-Retzius cells were increased in number (Imayoshi et al., 2008), suggesting a reciprocal regulation of these cell types. An involvement of Notch signaling in the development of the Zebrafish (*Danio rerio*) choroid plexus has also been described (Bill et al., 2008). Overexpression of Notch1, on the other hand (activated Notch1 in the Gdf7-lineage), massively increased the size of the mouse hindbrain choroid plexus (Hunter and Dymecki, 2007). Further evidence for the involvement of Notch in choroid plexus development includes the formation of choroid plexus tumors after E9 injection of a virus encoding constitutively active Notch3 (Dang et al., 2006). Additionally, alterations in Notch-receptor localization and expression have been found in human choroid plexus tumors when compared to normal choroid plexus tissue (Beschorner et al., 2013).

After choroid plexus specification, the choroid plexus anlage go through a characteristic thinning of the epithelium, going from pseudostratified to a proper single layer epithelium as it starts to invaginate into the ventricles (Dziegielewska et al., 2001). The majority of the choroid plexus epithelial cells are post-mitotic and the plexus grows into the ventricle through addition of cells from the proliferative zone at “the root” (Figure 3, Liddelow et al., 2010), which adds to the size of the choroid plexus through-out development. The choroid plexuses go through several stages of morphologically characterized development before reaching maturity postnatally (Dziegielewska et al., 2001).

The stroma of the choroid plexus (mesoderm derived, Catala, 1998), is induced by the choroid plexus anlage. Using chimeric transplantation experiments between chick and quail embryos, it was shown that the choroid plexus anlage (i.e., yet without stroma) from quail transplanted into the gut of a chick embryo induced the formation of a morphologically normal choroid plexus with organotypic fenestrated blood-vessels (Wilting and Christ, 1989). The blood vessels and the stroma were found to be of chick (host) origin demonstrating that the choroid plexus anlage induces the formation of the stroma and the blood vessels therein. In a different experiment they also showed that transplanting a non-choroid plexus forming neural epithelium to the normal site of choroid plexus development, in contact with the prospective stroma, did not induce choroid plexus characteristics (Wilting and Christ, 1989). However, even though neither the stroma nor the blood vessels induce choroid plexus formation, signaling from the stroma to the epithelial cells after stromal formation has been observed. This was seen recently when Shh was deleted from the choroid plexus epithelial cells, which lead to a decrease in Shh-signaling in the Shh responsive cells (choroid plexus progenitor cells and pericytes), which in turn resulted in a much smaller choroid plexus deficient in both vasculature and CPE cells (Huang et al., 2009; Nielsen and Dymecki, 2010).

Interestingly, this also leads us to one of the very few described regional differences between the choroid plexuses; *Shh* is not expressed in the forebrain choroid plexuses, suggesting that other molecules might be responsible for this regulation in the forebrain. Conversely, it appears that Otx2, which is expressed in and has an effect on the initial development of all the choroid plexuses, may have alternative functions in the different choroid plexuses after their specification and initial development, as deletion at E15 only affected survival in the hindbrain choroid plexus (Johansson et al., 2013). Further comparisons between the different choroid plexuses are currently under investigation.

## Regulation of CSF secretion and composition during development

Three main components are required for fluid transport/secretion across an epithelium: (i) free passage of water and solutes between the cells has to be inhibited via the presence of tight-junctions (i.e., a barrier), (ii) the capacity of water transport across the cells (i.e., the presence of water channels), and (iii) the presence of transporters and exchangers that can create an ionic or osmotic gradient that drives water across the epithelium. These criteria are of course all met in the adult choroid plexus via the presence of the blood-CSF barrier, the presence of the water channel AQP1 and the creation of ion gradients between blood and CSF via a multitude of ion transporters and exchangers (see e.g., Wolburg and Paulus, 2010). In development the barrier criterion is met by the presence of continuous tight junctional strands as soon as the choroid plexus invaginates into the ventricles (Møllgård and Saunders, 1975; Møllgård et al., 1976; Ek et al., 2003; Johansson et al., 2006). Aquaporin-1, the only confirmed and consistently identified water channel in the choroid plexus, is present in the choroid plexus epithelial cells as soon as they start to differentiate (Johansson et al., 2005), making water transport possible from very early stages in choroid plexus development. The gradient, however, that drives the water transport, does not appear to be regulated in the same manner as in the adult (Johansson et al., 2008a,b; Liddelow et al., 2013). For example, two of the main components in creating the ion gradients in the adult (NaK-ATPase alpha subunit and carbonic anhydrase II) are not present in the early developing choroid plexus (Johansson et al., 2008b). Instead it has been suggested that the there is a colloid-osmotic gradient, created by the high protein concentration in CSF during development, that drives the water from blood (or more accurately from interstitial fluid) into CSF (see Johansson et al., 2008a). Experimental evidence for this suggestion was recently described, where the total protein concentration in CSF was artificially increased via systemic protein injections in early development (P9 *Monodelphis Domestica*), resulting in a transient doubling of the size of the lateral ventricles (Liddelow et al., 2011a).

In both development and in the adult little is known about the transcriptional regulation of CSF secretion. One transcription factor that appears to have a role in regulating CSF secretion is E2F5, which is expressed in the developing choroid plexus from E12.5 (Swetloff and Ferretti, 2005). Its deletion (homozygous full knock-out mice) resulted in post-natal non-obstructive hydrocephalus (Lindeman et al., 1998), suggesting that it normally suppresses secretion. However, as E2F5 protein levels appear to decrease in the choroid plexus as they mature (and its localization is shifted from nuclear to cytoplasmic), it was suggested that E2F5 plays a role in the maturation process of CPE cells (Lindeman et al., 1998). It was also found that E2F5 is one of the target genes of choroid plexus specific micro-RNA mir449, which is expressed through-out choroid plexus development (Redshaw et al., 2009), suggesting a yet unexplored role of microRNAs in choroid plexus function, CSF secretion and potentially in the regulation of neurogenesis.

## The impact of CSF pressure and composition on neurogenesis

The continuous CSF production (originating mostly from the choroid plexuses) creates a pressure inside the cerebral ventricles. The requirement of this pressure for normal brain development was shown by a study in chick embryos (Stage18), where the intraventricular pressure was removed via insertion of hollow tubes into the ventricular system (Desmond and Jacobson, 1977). After 24 h the nervous tissue had folded into the ventricles. The ventricles themselves were smaller (only 20% of their normal size) and the surrounding brain tissue was not only disorganized but also reduced in size (Desmond and Jacobson, 1977).

However, the choroid plexuses not only regulate the level of CSF production but also modify its composition. CSF composition can be regulated in two main ways; either via changes in the transfer of molecules from blood into CSF across the epithelium (receptor or carrier mediated transfer as well as exclusion of molecules via efflux pumps) or via changes in the secretion of molecules produced by the choroid plexuses themselves (Figure 2). In the developing choroid plexuses both of these mechanisms are in play. The subset of epithelial cells that transport proteins from blood into CSF are not only present in development but their proportion is larger and they appear to be more specialized as they selectively transport certain plasma proteins and not others across the barrier, a mechanism that appears to not be present in the adult (for example see Johansson et al., 2008a; Liddelow et al., 2011b; 2013). In regards to other proteins capable of modifying CSF composition in the adult (efflux pumps, amino acid transporters, solute transporters), the majority are already present in the developing (e.g., E15 rat) choroid plexus but are often expressed at different levels (both higher and lower) compared to the adult (Ek et al., 2010; Kratzer et al., 2013; Liddelow et al., 2013) again suggesting a dynamic and regulated mechanisms of CSF composition control. In addition, recent choroid plexus transcriptome analyses have shown that the developing choroid plexuses also express a large amount of signaling type of molecules that can be secreted into the ventricles and affect the behavior of the neural stem cells lining the ventricles (e.g., Johansson et al., 2013, Table 1).

That CSF can modulate brain development has been shown in *in vitro* experiments. Here it was shown that isolated cortical cells can be maintained on embryonic CSF alone (Miyan et al., 2006). Neuroepithelial explants from chick were shown to survive and proliferate just as well in CSF serum free media as in serum supplemented media (Gato et al., 2005). Further, CSF from different ages affects the CSF differently, as seen in rat cortical cultures where E19 cells proliferated more in CSF from E19 and E20 compared to CSF from E18 and E21 (Miyan et al., 2006). Similar results were shown with E14 rat neurospheres, which, when cultured with E17 CSF, generated more spheres than when grown in CSF from E14, P6 or adult CSF (Lehtinen et al., 2011). This is consistent with the age-dependent changes in CSF composition previously observed using both proteomics (Zappaterra et al., 2007), total protein measurements (see Johansson et al., 2008a) and individual protein measurements (Dziegielewska et al., 1981; Huang et al., 2010; Lehtinen et al., 2011). The proteome study compared embryonic mouse CSF from three different ages and reported 423 proteins in E12.5 CSF, 318 in E14.5 and 382 in E17.5 CSF (all sampled from the lateral ventricle). Only 137 proteins were common to all three ages, suggesting a highly dynamic regulation of CSF composition (Zappaterra et al., 2007). In studies investigating particular molecules, the levels of different plasma derived proteins was found to vary between different embryonic stages and although the individual proteins were following the same pattern (increase, peak, and decrease) the peak was reached at different ages (Dziegielewska et al., 1981). Igf2 was found in CSF from embryonic rat at all ages investigated but showed a significant increase late in embryogenesis and a decrease again perinatally (Lehtinen et al., 2011). However, in a study measuring Shh levels, the concentration of this particular molecule did not change in mouse CSF between E12.5 and E15.5 (Huang et al., 2009). Together these studies show a highly regulated control of CSF composition during development and during the period of neurogenesis.

In support of the regulation of CSF content having a physiological function, direct manipulation of CSF composition has been shown to alter the behavior of the stem cells in direct contact with the CSF. Here, both loss and gain of function experiments have been performed *in vivo* using either neutralizing antibodies or recombinant proteins injected into the embryonic ventricles. For example, the removal of Fgf2 from chick CSF caused a loss of proliferation and increased neural differentiation (Martin et al., 2006). Similar experiments with another type of growth factor Igf1, in embryonic mice and rats, showed an increase in proliferation after intraventricular injections of recombinant proteins and a decrease after IGF-neutralizing antibodies were injected (Mairet-Coello et al., 2009). Thus, the link between CSF composition and alteration in neural stem cell behavior has been experimentally shown but the link between choroid plexus development and brain development remains less clear.

## The specific impact of the choroid plexuses on developmental neurogenesis

The choroid plexuses appear whilst neurogenesis is occurring through-out the nervous system. This gives the potential for the choroid plexuses (via their modulation of CSF) to alter the behavior of the neural stem cells along the neuroaxis (Figure 1, Box 1). As described above, the capability of the CSF to alter both proliferation and survival in different brains regions *in vitro* has long been known, likely due to the signaling molecules present in CSF. However, to what extent these molecules are regulated or even secreted by the choroid plexuses has not been sufficiently investigated (perhaps in part due to the technical difficulties such an approach poses).

With the advancement of region and time specific deletion techniques new strategies to isolate choroid plexuses involvement in neurogenesis are available. Along these lines, some recent work has demonstrated, via choroid plexus specific deletions, that molecules expressed in the choroid plexus have the capability to influence brain development at other sites. This was first achieved by deletion of Shh from the hindbrain choroid plexus (using the Wnt1-Cre), resulting in a more than 50% decrease in the proliferation of the neural stem cells in the nearby cerebellum and impaired GABAergic progenitor expansion (Huang et al., 2010).

Recently, in work done by the author and colleagues, it has also been shown that when the transcription factor Otx2 was deleted from the hindbrain choroid plexus using the Gd7-Cre (only very sparse recombination in other regions) it not only significantly altered the size of the choroid plexus but also the relative expression of many secreted signaling molecules (Johansson et al., 2013). To our surprise, this deletion led to a region-specific increase in proliferation in the far distant cerebral cortex (but not in the spinal cord or in the lateral ganglionic eminence), which in turn led to site-specific alterations in the cortical neuronal layers at P7. The increase in proliferation was caused by an increase in Wnt-signaling, mediated by, at least in part, altered hindbrain choroid plexus expression levels and the ensuing increased CSF protein levels of the Wnt-signaling modulators Wnt4 and Tgm2.

**Figure 4:**
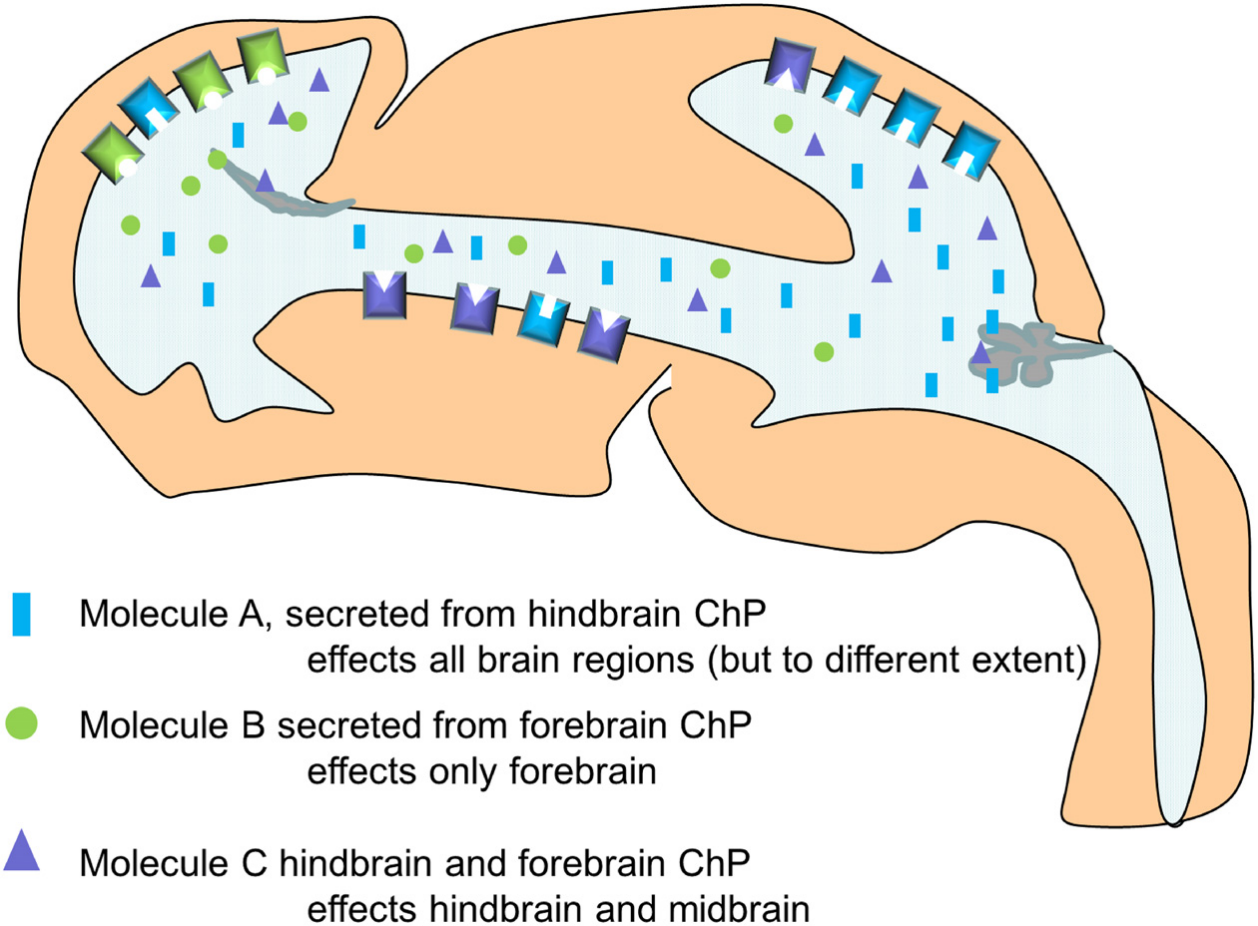
**Simplified schematic drawing of how choroid plexus mediated changes in CSF composition differentially alters the behavior of the neural stem cells along the neuroaxis via differences in their ability to respond (here exemplified by the presence and density of receptors)**.

This work revealed for the first time, the choroid plexus as a potential modulator of Wnt-signaling in the developing brain. It also highlighted that the hindbrain choroid plexus, being the first to appear, can alter the behavior of progenitor cells at the opposite end of the ventricular system prior to the differentiation of the other choroid plexuses. This period is also notable as the ventricular system is still an enclosed cavity, not connected with the fluid in the subarachnoid space and does not yet have any directional flow, making it more possible for molecules secreted from the fourth ventricular choroid plexus to reach the forebrain and influence the behavior of the neural stem cells (Figure 4). In support of this, in the above study we also found that the increase in proliferation and Wnt-signaling was no longer present at E16, when the influence of the hindbrain choroid plexus would be much reduced due to the now much larger (unaffected) lateral ventricular choroid plexuses in closer proximity to the cortical neural stem cells (Johansson et al., 2013).

This work also showed that not all regions in the CNS responded to the altered CSF in the same way (or at all). We suggest that select tissues are prepared for certain signaling molecules, by the expression of not only receptors but also by the expression of modulators and inhibitors of the different signaling pathways. Thus, neurogenesis can be seen as a reciprocal process between the choroid plexus and the neural stem cells mediated via the CSF (Figure 4).

## Conclusions and future directions

In conclusion, there is substantial evidence that there is indeed a role played by the choroid plexus-CSF-signaling axis in the modulation of developmental neurogenesis. However, much more remains to be discovered. The specific mechanisms by which the choroid plexuses modulate brain development both in terms of the choroid plexuses as whole organs as well as specific aspects of their functions (e.g., transfer of blood-borne molecules or secretion of a particular molecule) needs to be investigated. Further detailed studies of choroid plexus impact on different brain regions and developmental time-points would increase our understanding of the whole process of brain development. Understanding the role of the choroid plexus-CSF signaling axis and its impact on brain development can also lead to novel routes and mechanisms for treatment approaches for a multitude of developmental disorders and in the long-run also for application to adult neurodegeneration and brain injuries.

### Conflict of interest statement

The author declares that the research was conducted in the absence of any commercial or financial relationships that could be construed as a potential conflict of interest.
